# Psychological Distress and Post-Traumatic Stress Symptoms among Medical Students in Vietnam: Associations with Non-Contact and Contact Sexual Violence Exposure

**DOI:** 10.64898/2026.07.25.26358649

**Published:** 2026-07-29

**Authors:** Phan Thi Minh Ngoc, Hoang Thi Hai Van, Tran Quynh Anh, Leah E. Daigle, Doan Ngoc Thuy Tien, Kathryn M. Yount

## Abstract

**Background:**

Medical students face significant mental health challenges, yet psychological distress and post-traumatic stress remain insufficiently characterized in lower-resource settings like Vietnam. Among potential stressors, sexual violence (SV) exposure represents a critical but understudied risk factor. This study evaluated the severity of psychological distress (PD) and post-traumatic stress symptoms (PTSS) among medical students in Northern Vietnam and examined their associations with past-year non-contact and contact SV exposure.

**Methods:**

A cross-sectional survey was conducted in May 2025 using REDCap among a probability sample of 555 medical students (years 2–5). Mental health outcomes were evaluated using validated instruments: the 21-item Depression, Anxiety, and Stress Scales (DASS-21) for past-week PD and the PTSD Checklist for DSM-5 (PCL-5) for past-month PTSS. Past-year non-contact and contact SV exposures were measured using the Sexual Experiences Survey–Victimization (SES-V). Multivariable linear regression modeled adjusted associations between SV exposure types and mental health symptom severity, testing for sex-specific differences.

**Results:**

Overall mental health symptom scores escalated significantly with SV exposure. Median PTSS scores increased from 11 among unexposed students to 20 for non-contact SV and 30 for contact SV ( *p* < 0.001). Similarly, median PD scores increased from 7 (unexposed) to 11.5 (non-contact SV) and 21 (contact SV) ( *p* < 0.001). Adjusted linear models demonstrated robust associations where both non-contact and contact SV exposure were strong independent predictors of heightened PD and PTSS severity, with no significant moderation by sex. In terms of exposure prevalence, 21.26% of students reported non-contact SV (higher in females: 26.24% vs. males: 16.12%) and 10.66% reported contact SV.

**Conclusions:**

Vietnamese medical students exposed to sexual violence experience a heavy mental health burden marked by elevated psychological distress and post-traumatic stress symptoms. To protect student mental health in lower-resource settings, medical institutions must establish comprehensive trauma-informed psychological support, confidential counseling services, and targeted intervention strategies addressing both contact and non-contact stress exposures.

**Highlights:**

- Prior-year non-contact and contact sexual violence are common in medical students in Northern Vietnam.
- Non-contact sexual violence is more common among female than male students.
- Contact sexual violence is more common among sexual-minority than heterosexual students.
- Non-contact and especially contact sexual violence are positively associated with psychological distress and post-traumatic stress.
- Inclusive, preventive and response services are needed to address sexual violence and its psychological impacts.

## Full Text Availability

The license terms selected by the author(s) for this preprint version do not permit archiving in PMC. The full text is available from the preprint server.

